# Therapeutic Targets for Neurodevelopmental Disorders Emerging from Animal Models with Perinatal Immune Activation

**DOI:** 10.3390/ijms161226092

**Published:** 2015-11-27

**Authors:** Daisuke Ibi, Kiyofumi Yamada

**Affiliations:** 1 Department of Chemical Pharmacology, Faculty of Pharmaceutical Sciences, Meijo University, 150 Yagotoyama, Tenpaku-ku, Nagoya 468-8503, Japan; ibid@meijo-u.ac.jp; 2 Department of Neuropsychopharmacology and Hospital Pharmacy, Nagoya University Graduate School of Medicine, Nagoya 466-8560, Japan

**Keywords:** schizophrenia, autism, animal model, polyI:C, astrocyte, interleukin-6, interferon-induced transmembrane 3, matrix metalloproteinase 3, gut microbiome

## Abstract

Increasing epidemiological evidence indicates that perinatal infection with various viral pathogens enhances the risk for several psychiatric disorders. The pathophysiological significance of astrocyte interactions with neurons and/or gut microbiomes has been reported in neurodevelopmental disorders triggered by pre- and postnatal immune insults. Recent studies with the maternal immune activation or neonatal polyriboinosinic polyribocytidylic acid models of neurodevelopmental disorders have identified various candidate molecules that could be responsible for brain dysfunction. Here, we review the functions of several candidate molecules in neurodevelopment and brain function and discuss their potential as therapeutic targets for psychiatric disorders.

## 1. Introduction

Abnormalities in early brain development contribute to the etiology of many neuropsychiatric disorders in later life [1,2,3,4]. Recent advances in genome-wide analysis indicate that large numbers of common variants shape individual disease risks, including those for mental illnesses [5,6,7]. However, the biological mechanisms by which environmental factors affect brain development are poorly understood. Environmental insults include maternal stress, nutritional deficiencies, perinatal infections, season of birth, and obstetric complications [8,9]. Several lines of epidemiological evidence suggest that prenatal infection and postnatal central nervous system (CNS) infection with various pathogens such as viruses, bacteria, and protozoan parasites enhance the risk for several neurodevelopmental disorders including schizophrenia [10,11,12,13,14,15] and autism spectrum disorder (ASD) [16,17]. These findings indicate the possible interference in brain development triggered by perinatal immune activation. Recent studies with animal models have identified various candidate molecules that could be responsible for the brain dysfunction after perinatal immune activation. Here, we discuss and review the potential therapeutic targets for drug discovery for neurodevelopmental disorders such as ASD and schizophrenia.

## 2. Endophenotypes of Animal Models with Perinatal Immune Activation

Based on epidemiological studies [10,11,12,13,14,15], the adult offspring of influenza virus-infected dams have been developed as an animal model of neurodevelopmental disorders, which exhibit some behavioral abnormalities [13,18]. Alternatively, to investigate the effect of immune-inflammatory responses on fetal brain development following viral and bacterial infection in pregnancy, pregnant rodents were injected with polyriboinosinic polyribocytidylic acid (polyI:C) and lipopolysaccharide (LPS), respectively. PolyI:C is recognized by a toll-like receptor (TLR) 3 as viral double-stranded RNA, which is produced during viral infection as a genetic replication for single-stranded RNA or as a secondary transcript of DNA viruses [19], and activates the innate immune system [20]. The offspring of pregnant mice or rats treated with polyI:C displayed behavioral abnormalities such as augmentation of psychostimulant-induced hyperactivity and impairments of social interaction, prepulse inhibition (PPI), and memory [21,22,23,24,25,26,27,28], which are considered behavioral phenotypes, corresponding to some domains of positive and negative symptoms, and cognitive dysfunction in patients with schizophrenia [29]. Furthermore, the offspring after maternal polyI:C treatment reproduce the neuropathological features characteristic of schizophrenia [30,31,32], which include enlarged lateral ventricles, reduced dendritic spine density, decreased dopamine D1 and metabotropic glutamate 2 receptors, and increased 5-HT_2A_ receptors in the prefrontal cortex, loss of parvalbumin in the hippocampal interneurons, and enhanced tyrosine hydroxlyase in the striatum [28,33,34,35]. The underlying mechanisms for behavioral and neuropathological deficits in the offspring caused by maternal immune activation (MIA) by polyI:C treatment are not fully understood, but evidence suggests the involvement of both pro-inflammatory and anti-inflammatory cytokines [25,27]. Systemic immune activation in dams characterized by increased levels of cytokines is supposed to compromise the placental barrier, allowing the entrance of maternally derived cytokines into the fetal circulation and inducing inflammatory responses in the fetal brain, which leads to developmental disturbances of fetal brain [17,33]. Some serious postnatal viral infections in the CNS have also been associated with the etiology of neurodevelopmental disorders [3,36,37,38]. Our initial study demonstrated that neonatal polyI:C treatment during postnatal days 2–6 causes brain dysfunction in adulthood, characterized by impairments of memory and social behaviors, PPI deficit, and impairment of hippocampal glutamatergic neurotransmission [39]. In addition, neonatal polyI:C treatment induces the neuropathological abnormalities such as reductions of spine density and dendrite complexity of cortical pyramidal neurons, as well as the downregulation of cortical MAP2 protein levels [40].

## 3. Astrocyte as a Possible Therapeutic Target

Glial cells are major cell populations in the CNS, which comprise oligodendrocytes, astrocytes, microglia, and NG2-positive cells [41]. Astrocytes, the most numerous glial cells in the CNS, are involved in the maintenance of efficient neurotransmission by the supply of energy metabolites, the turnover of neurotransmitters, and the release of gliotransmitters such as glutamate and d-serine as well as establishing the blood-brain barrier (BBB) [42,43,44]. Accumulating evidence also shows that astrocytes play a key role in normal brain development [45,46]. Notably, researchers have demonstrated the indispensable nature of the contribution of astrocytes to neuronal development and synaptic formation [47,48,49], some of which can be explained by neuron–glia interactions through the humoral factors secreted from astrocytes [41,44,50,51,52,53]. In recent years, several families of astrocyte-secreted molecules have been identified [54,55,56,57,58,59] and shown to regulate several aspects of neuronal development and synaptic formation. Meanwhile, astrocytes also coordinate immune response to invading pathogens and peripheral inflammatory factors [60]. In fact, it has been reported that thousands of molecules are released from astrocytes responding to pro- and anti-inflammatory cytokines and TLR ligands such as bacterial LPS and specific nucleic acids like polyI:C [40,61,62,63,64,65]. Astrocytes exposed with maternally derived cytokines after MIA seem to secrete various cytokines that affect brain development. Regarding TLRs expressed in astrocytes, it is noteworthy that TLR 3 is the predominant one [40,63,64], given that the basal expression levels of TLR 3 in neurons and microglia are even lower than in astrocytes [40,63]. PolyI:C and viral nucleic acids supposedly target the TLR 3 of astrocytes in either the brain or the BBB. We have previously demonstrated that the humoral factors released from astrocytes stimulated with polyI:C disrupt neuron–glia interactions, resulting in neurodevelopment impairment [40,63]. In addition, CNS infection by the protozoan parasite toxoplasma gondii is suggested to affect brain development and function though the humoral factors released from astrocytes [15,66]. According to observations in postmortem brains, morphological and functional abnormalities of astrocytes have been reported in patients with neurodevelopmental disorders such as ASD and schizophrenia [67,68,69], raising a possibility that astrocyte-secreted factors in these brains contributed to neurodevelopmental impairments. These possibilities led us to discuss possible therapeutic approaches for neurodevelopmental disorders by targeting astrocytes and/or astrocyte-secreted molecules.

## 4. Interleukin-6

Interleukin-6 (IL-6), identified as a B-cell differentiation factor [70], is a major pro-inflammatory cytokine involved in immune system-brain interplay [2]. IL-6 has been implicated in stress responses, synaptic plasticity, cognition, sleep, and neurodevelopment [2,71]. Altered levels of IL-6 in the cerebrospinal fluid have been found in patients with schizophrenia and ASD [72,73]. Smith and colleagues demonstrated that IL-6 derived from dam following maternal polyI:C treatment mediates the effects of MIA on the brain development of the fetus [27]. An injection of IL-6 in pregnant mice also results in the development of impairments in PPI and latent inhibition in the adult offspring [27], both of which are linked to schizophrenia and ASD [74,75,76,77]. They also showed that IL-6 inhibition through antibody application or knockout (KO) dam use significantly ameliorates such cognitive impairments in adult offspring following MIA [27]. These results suggest that IL-6 is involved in the effects of MIA on fetal brain maturation, raising a possibility that IL-6 and its receptor may be potential targets for drug discovery in neurodevelopmental disorders. However, it should be noted that IL-6 may also behave as a mediator of neuroprotection and neurotrophy in different settings. For example, in various brain injury models, IL-6 deficiency induces delayed reactive astrocyte/astrogliosis and increased BBB permeability, and the IL-6-induced astrogliosis following trauma is important in restoring BBB integrity and repairing the lesion [64,78,79]. Several data in cultured astrocytes demonstrate that IL-6 produces neuroprotective and neurotrophic factors including the nerve growth factor, neurotrophin-3, and neurotrophin-4/5 [80]. Similarly, polyI:C is known to activate the production and secretion of IL-6, as well as neuroprotective and neurotrophic factors in cultured astrocytes [64,81,82], suggesting that astrocytes stimulated by polyI:C play a role in the neuroprotective innate immune response though the IL-6 system [82]. Given that astrocyte-specific deletion of either IL-6 or its receptor causes behavioral abnormalities [83], it may be necessary to fully understand where, when, and how IL-6 and/or its receptor express and function for normal brain development.

## 5. Interferon-Induced Transmembrane 3

Interferons (IFNs) prevent the incursion of viral genomes from the endosomal cascade, and the induction of interferon-induced transmembrane (IFITM) 3 is essential for this function. Thus, mice with IFITM3 deficiency (IFITM3 KO mice) exhibit accelerated progression, and higher mortality as well as systemic viral burdens following influenza A virus exposure [84,85,86,87]. IFITM3 plays an important role in cellular defense against the infection of various viruses such as influenza A, HIV-1, dengue, filoviruses, and coronaviruses [85,87,88,89,90,91,92]. The role of IFITM3 in the CNS has not yet been fully elucidated, although its increased expression was demonstrated in the brains of patients with neuropsychiatric diseases [93,94,95,96,97,98]. A transient expression of IFITM3 in the brains of mice treated with polyI:C happened only in astrocytes and not in neurons or microglia. PolyI:C treatment in neonatal wild-type mice results in deficits of cognitive and social behaviors, activity-dependent glutamate release in the hippocampus, and morphological maturation of dendrites and spines of cortical neurons in adulthood, all of which are preserved in IFITM3 KO mice [40]. The humoral factors derived from astrocytes exposed to polyI:C impair the dendrite elongation and spine formation of cultured neurons *in vitro*, but this effect of polyI:C is abolished when using astrocytes derived from IFITM3 KO mice. These findings suggest that the induction of IFITM3 in astrocytes during neurodevelopment has non-cell autonomous effects that in turn impair neurodevelopment via the disruption of neuron–glia interaction [40]. Importantly, the up-regulation of IFITM3 mRNA in the fetal brain was confirmed following MIA with polyI:C treatment. Pregnant mice received once-daily intravenous injections of polyI:C on days 16 and 17 of pregnancy, and the fetal cortices and hippocampi were removed 12 h after the final injection on day 17. MIA increased IFITM3 mRNA levels to 177% ± 40% in the cortex and 190% ± 20% in the hippocampus as compared to the levels in vehicle-treated control offspring. Given that IFITM3 expression is up-regulated in the brains of patients with schizophrenia [93,94,95] and ASD [96], it is proposed that IFITM3 might be a novel drug target for the treatment of these neurodevelopmental disorders [40,95]. However, because IFITM3 plays an important role in cellular defense against virus infection, it is necessary to ensure that IFITM3 targeting in astrocytes has no effect on the cellular defense system against virus infection. Type I IFNs are known to be strong inducers of not only IFITM3 but also IL-6 [27,40,81,99]. Together, type I IFN-signaling cascades to induce IFITM3 and IL-6 may be possible targets for neurodevelopmental disorders triggered by perinatal immune activation.

## 6. Matrix Metalloproteinase 3

Matrix metalloproteinases (MMPs) are known to have deleterious roles in various nervous system diseases [100,101,102,103,104,105,106]. In particular, MMP 3 degrades the extracellular matrix (ECM) such as integrins, *N*-syndecan, and non-ECM proteins and activates other MMP subtypes [107,108,109,110,111,112,113]. Up-regulated MMP 3 in the brain following neuronal damage and inflammation is thought to underlie the pathophysiology of neurologic and neuropsychiatric diseases [105,114,115,116,117,118]. Notably, associations have been reported between schizophrenia and Alzheimer’s disease of polymorphism on the MMP3 promoter, which seems to regulate the transcriptional activity [119,120], raising a possibility that the alternation of MMP3 expression may be involved in the etiology of these diseases. We have identified MMP3 as a humoral factor released by astrocytes following polyI:C treatment, by which the dendritic development of cultured neurons is partially but significantly impaired *in vitro* [63]. These results suggest that MMP3 released by astrocytes disrupts the neuron–glia interaction, leading to neurodevelopmental impairments. Hence, further studies are required to elucidate which MMP3 substrates contribute to the impairment of neuronal development. Understanding the molecular pathways involved in these events would provide insight into novel therapeutic approaches to neurodevelopmental disorders following perinatal viral infections.

## 7. Brain–Gut Interaction

Recent preclinical investigations have indicated that gut microbes have a significant effect on brain function [121,122,123], which is mediated by the neuroactive factors generated by such bacteria. The biological diversity of this biogeocenosis is established early in life and is highly affected by environment [121]. Alternated communications between such microbiome and the brain seem to be involved in neurodevelopmental disorders, a fact that has recently received considerable attention [124,125,126,127]. Recent clinical studies have demonstrated that gastrointestinal disturbances are a major comorbidity in ASD patients [125,126]. Furthermore, alteration in the composition and metabolites of the gut microbiome has long been considered a possible mechanism contributing to ASD pathophysiology, a hypothesis that is supported by the recent findings using MIA offspring [124]. The offspring of pregnant mice with MIA by polyI:C treatment exhibit alterations in gut physiology, microbial composition, and profile of metabolic products [124]. The observed abnormal behaviors in the MIA offspring are relieved by a bacteroides fragilis probiotic [124], suggesting that microbiome-mediated therapies may be a safe and effective treatment for ASD and other neurodevelopmental disorders. One of the transmissions of the gut state seems to be communicated through the circulatory system [125,126,127,128] by which cytokines and bacterial metabolites derived from the gut may activate BBB astrocytes [128]. Interestingly, the elevated IFITM3 mRNA expression has been demonstrated in vascular endothelial cells of the BBB in the brains of patients with schizophrenia [95,129]. Understanding the role of astrocytes and/or the BBB in the brain–gut microbiome interactions may provide new possible therapeutic approaches to neurodevelopmental disorders.

## 8. Conclusions

**Figure 1:**
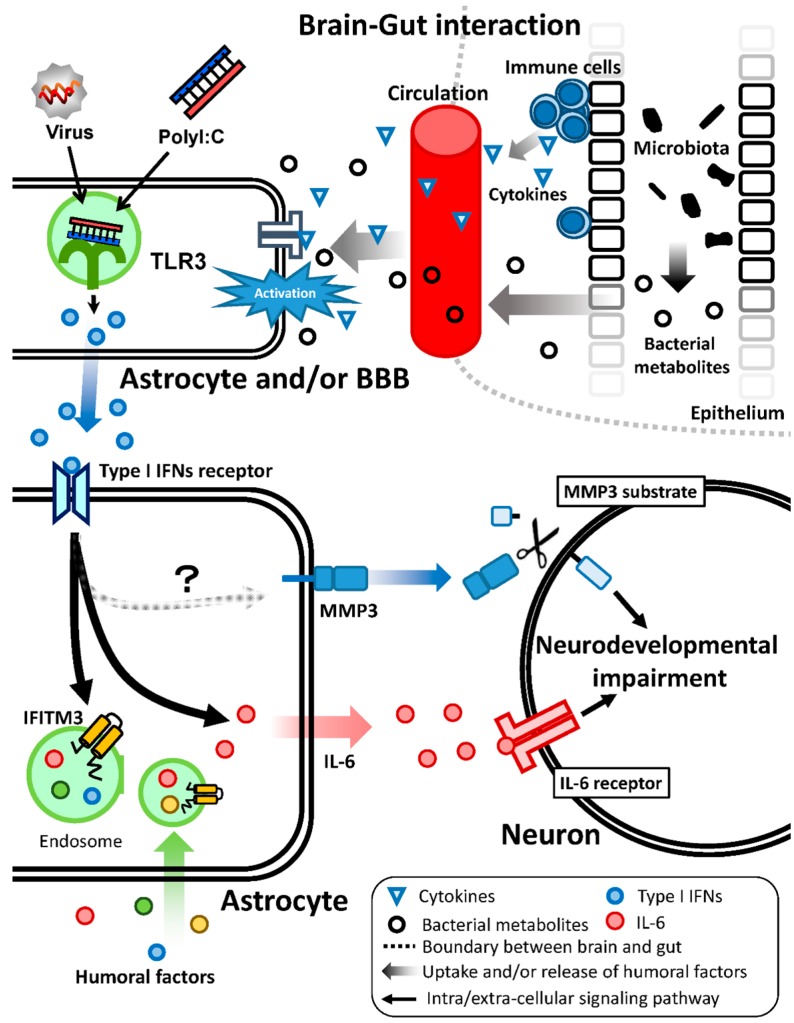
This schematic presents the model for neurodevelopmental disturbances by polyI:C treatment. PolyI:C induces type I IFNs expression in astrocytes and/or the BBB; subsequently, the released type I IFNs induce various molecules in an autocrine and paracrine manner, some of which, such as IFITM3 and IL-6, disrupt neuron–glia interaction, affecting neurodevelopment and in turn, brain functions.

We propose a model for neurodevelopmental impairments though the disruption of the neuron–glia interaction after polyI:C treatment (Figure 1). Viral double-stranded RNA, as well as polyI:C, activate TLR 3 in astrocytes of the brain parenchyma or BBB. Cytokines and bacterial metabolites from the gut may also activate BBB astrocytes. Type I IFNs are produced and released by these activated cells. IFITM3 and IL-6 are induced in astrocytes in a paracrine and/or autocrine manner. MMP3 is also induced by polyI:C-treated astrocytes even if the mechanisms of MMP3 induction in astrocytes remain to be determined. Thereafter, the secreted IL-6 and soluble MMP3 from astrocytes would access neurons and affect neuronal development and functions though the activation of IL-6 receptors and degradation of MMP3 substrates, respectively. Additionally, the endosomal IFITM3 in astrocytes modulates their endocytic activity, which may affect the extracellular levels of humoral factors. These abnormalities of the humoral factors, including IL-6 and soluble MMP3 after polyI:C treatment, impair neuronal development, resulting in psychiatric disorders such as schizophrenia and ASD.
